# Neuroimaging in cluster headache and other trigeminal autonomic cephalalgias

**DOI:** 10.1007/s10194-011-0403-8

**Published:** 2011-11-25

**Authors:** Elisa Iacovelli, Gianluca Coppola, Emanuele Tinelli, Francesco Pierelli, Federico Bianco

**Affiliations:** 1 Neurology Section, Department of Medico-Surgical Sciences and Biotechnologies, “Sapienza” University of Rome, Viale dell’Università 30, 00185 Rome, Italy; 2 Department of Neurophysiology of Vision and Neurophthalmology, G.B. Bietti Eye Foundation-IRCCS, Rome, Italy; 3 Department of Medico-Surgical Sciences and Biotechnologies, “Sapienza” University of Rome, Polo Pontino, Latina, Italy; 4 IRCCS-Neuromed, Pozzilli (IS), Italy

**Keywords:** Trigeminal autonomic cephalalgias, Hypothalamus, Pain neuromatrix, Single photon emission computed tomography, Positron emission tomography, Magnetic resonance spectroscopy, Functional MRI, Voxel-based morphometry

## Abstract

The central nervous system mechanisms involved in trigeminal autonomic cephalalgias, a group of primary headaches characterized by strictly unilateral head pain that occurs in association with ipsilateral craniofacial autonomic features, are still not comprehensively understood. However, functional imaging methods have revolutionized our understanding of mechanisms involved in these primary headache syndromes. The present review provides a brief overview of the major modern functional neuroimaging techniques used to examine brain structure, biochemistry, metabolic state, and functional capacity. The available functional neuroimaging data in cluster headache and other TACs will thus be summarized. Although the precise brain structures responsible for these primary headache syndromes still remain to be determined, neuroimaging data suggest a major role for posterior hypothalamus activation in initiating and maintaining attacks. Furthermore, pathophysiological involvement of the pain neuromatrix and of the central descending opiatergic pain control system was observed. Given the rapid advances in functional and structural neuroimaging methodologies, it can be expected that these non-invasive techniques will continue to improve our understanding into the nature of the brain dysfunction in cluster headache and other trigeminal autonomic cephalalgias.

## Introduction

Trigeminal autonomic cephalalgias (TACs), such as cluster headache (CH), paroxysmal hemicrania (PH) and short-lasting unilateral neuralgiform headache attacks with conjunctival injection and tearing (SUNCT) are a group of primary headaches which share the activation of the trigeminal nerve in mediating pain and of the parasympathetic component of the seventh cranial nerve in producing the local autonomic symptoms [1]. However, the CNS mechanisms involved in these primary headaches are still not comprehensively understood.

Modern methods of functional neuroimaging have revolutionized our way of exploring brain function, and their application in the study of TACs has provided great advances in the understanding of their pathophysiological facets.

Here, we will provide first a brief overview of the neuroimaging techniques used to study brain function in TACs. Thereafter, we will review the knowledge acquired with these functional neuroimaging methods on the pathophysiology of primary headaches.

## Neuroimaging techniques

While early studies in trigeminal autonomic headache were done exclusively with positron emission tomography (PET), the development of new imaging techniques as well as the increase in image resolution have led to an expansion in the applications of magnetic resonance (MR) based imaging methods in headache. Both PET and MR-based imaging methods have now been widely used in trigeminal autonomic headache research. As well as these imaging methods that enable us to investigate the function of the brain during headache attacks, structural and morphometric methods, such as voxel-based morphometry, have also become available. In contrast to conventional methods, voxel-based morphometry allows the comparison of the local concentration of gray matter between groups of subjects and the correlation of whole-brain gray matter density with clinical variables of the disease.

### Single photon emission computed tomography (SPECT)

First studies in headache research have applied SPECT, a procedure based on nuclear medicine imaging techniques. SPECT can be used to define a patient’s pathologic status when neurologic or psychiatric symptoms cannot be explained by structural neuroimaging findings [2]. Molecules labeled with a radioactive isotope are administered by IV injection. A positron-sensitive detector allows the detection of photon emissions. SPECT isotopes are chosen on the basis of their chemical, biologic, and nuclear properties [3]. The main characteristics include: a half-life long enough to allow tracer preparation, injection, and uptake and image acquisition and short enough not to deliver an unnecessary dose to the patient; gamma emission energy sufficiently high to escape from the patient, but sufficiently low to facilitate collimation and detection and favorable dosimetry to limit patient exposure [3]. The tracer and isotopes commonly used in brain SPECT are xenon-133 (^133^Xe), iodine-123 isopropyliodoamphetamine (^123^I-IMP), more recently replaced by technetium-Tc99m-labeled hexamethylpropyleneamine oxime (Tc99m-HMPAO) and Tc99m-ethyl cysteinate dimer (ECD). The brain perfusion imaging agents 99mTc-HMPAO and 99mTc-ECD are sensitive indicators of regional cerebral blood flow (rCBF) changes and can detect a reduction in blood flow [2].

### Positron emission tomography

In headache research many studies have applied PET. PET is a nuclear medicine imaging technique that permits to acquire images of physiologic function by studying cerebral blood flow as an indirect marker of neuronal activity. The scan uses a gamma-ray emitting radio-isotope bound to a biologically active molecule. The radiotracers principally used are 2-deoxy-2-[18F] fluoro-d-glucose (18FDG) [4, 5], ^15^O labeled water (H_2_^15^O) [6], or radioactively labeled ligands that bind to specific receptors [7, 8]. After seconds or few minutes following the injection or inhalation of the radiopharmaceutical, the radio-isotope decays emitting a positron, which moves a short distance before annihilating with an electron. The annihilation produces a couple of photons that are detected with a gamma camera. In modern scanners, three-dimensional images of tracer concentration within the body are then constructed by computer analysis, accomplished with the aid of a CT X-ray scan, performed at the same time. Radiopharmaceuticals most commonly used, such as 18FDG and H_2_^15^O, allow imaging of tissue metabolic activity, in terms of regional glucose uptake or blood flow changes, respectively. Imaging of the tissue concentration of other types of molecules of interest can be obtained by using different tracers.

### Magnetic resonance spectroscopy

Magnetic resonance spectroscopy (MRS) is based on the principle that the resonance frequency of a nucleus depends on its chemical environment, produces a small, but perceptible, change in the resonance frequency of that nucleus. This nuclear behavior is termed “chemical shift” and is caused by the magnetic fields generated by circulating electrons surrounding the nuclei, interacting with the main magnetic field. Protons (^1^H) have been traditionally used for MRS because of their high natural abundance in organic structures and high nuclear magnetic sensitivity compared with any other magnetic nuclei [9]. Moreover, diagnostically resolvable hydrogen MR spectra may be obtained with clinical instruments (1.5 T or greater) and routine coils. MR spectroscopy allows mapping the distribution of metabolite concentrations within small volumes of cerebral tissue, termed *volume of interest* (VOI) [10]. Using long echo-times (TEs), the signal from most metabolites in the brain is lost except for choline (Cho), creatine (Cr), *N*-acetyl aspartate (NAA), and lactate. NAA is accepted as a marker of neuronal density and viability. Cho is a metabolic marker of membrane density and integrity. The peak for Cr is a marker of energy metabolism. Lactate appears under conditions where the aerobic oxidation mechanism fails and anaerobic glycolysis takes over. Lipids reflect necrotic processes [11]. Moreover, other metabolites (e.g., myoinositol, glutamate, glutamine, and glycine) can be identified by using short TEs [12].

### Functional MRI

Functional MRI was used to generate the first functional magnetic resonance maps of human task activation. In 1990, Ogawa et al. [13] reported that MRI was sensitive enough to show “blood oxygenation level-dependent” (BOLD) signal changes in vivo. Since it was known that changes in neuronal activity were accompanied by local changes in brain oxygen content [14], it became evident that a technique based on the BOLD effect could potentially be used to investigate neuronal activation through changes induced in tissue oxygenation. The signal changes in BOLD fMRI are determined by the paramagnetic properties of deoxyhemoglobin (deoxy-Hb) [15]. According to the BOLD signal theory, these changes are determined by a marked increase in regional blood flow. Because blood oxygenation levels change rapidly following the activity of neurons in a brain region, fMRI allows researchers to localize brain activity on a second-by-second basis, and within millimeters of its origin. In addition to the BOLD effect, other mechanisms are supposed to contribute to the endogenous MR contrast. It is well known that inflow effects make some contribution to the functional signal change [16].

### Voxel-based morphometry

Voxel-based morphometry (VBM) permits to identify volume differences in brain anatomy between groups of subjects, performing statistical tests across all voxels in the image [17]. The procedure involves the acquisition of T1-weighted volumetric MRI scans that are registered onto the same template image so that they are all in the same space, to perform statistical analyses across multiple MRI scans from different individuals. This process is known as spatial normalization [18]. The spatially normalized images are next segmented into gray matter (GM), white matter (WM) and cerebrospinal fluid (CSF). Although both gray and white matter volumes can be assessed using VBM, the majority of VBM studies concentrate on gray matter [18]. The images, after the normalization and segmentation, are smoothed and processed to perform a parametric statistical analysis [17]. All voxels that refute the null hypothesis and show statistical significance generate statistical maps that are often shown as color maps with the scale representing the *t* statistic [18]. However, in more recent studies, the segmentation of images into GM, WM and CSF is first performed and then GM maps are applied a high-dimensional DARTEL normalization modulating for nonlinear effects [19, 20].

In the following section, we will summarize the results of functional imaging studies on trigeminal autonomic headache syndromes, showing how functional neuroimaging has advanced our understanding of trigeminal autonomic headache pathophysiology (Tables 1, 2).

**Table 1:** All neuroimaging findings in cluster headache and other trigeminal-autonomic cephalalgias

Reference	No. of subjects and diagnosis	Timing of the scan	Neuroimaging techniques	Main results
Norris et al. [23]	1 episodic CH	During the attack	SPECT	No differences in mean CBF
Sakai et al. [24]	9 episodic CH		SPECT	Increased CBF
Henry et al. [25]	3 episodic CH	Inside a bout during an attack	SPECT	No differences in mean CBF
Nelson et al. [26]	26 episodic CH	4 during the attack. 7 after nitroglycerine, alcohol, histamine or oxygen	SPECT	Patients showed a variable pattern of increased or decreased mean CBF
Krabbe et al. [27]	18 episodic CH	18 outside the attacks 8 during the attack	SPECT	No differences in mean CBF
Di Piero et al. [28]	7 episodic CH	Outside of a bout	SPECT	CBF lower in contralateral primary sensorimotor and thalamic regions compared to healthy subjects
Hsieh et al. [29]	7 episodic CH	4 during the bout 3 out of the bout (nitroglycerine-induced attacks)	PET	Increased rCBF in the right caudal and rostrocaudal ACC, temporo-polar region, supplementary motor area, bilaterally in the primary motor area, premotor areas, opercular region, insula/putamen, and lateral inferior frontal cortex rCBF lower in the bilateral posterior parietal cortex, occipito-temporal region and prefrontal cortex
May et al. [30]	9 chronic CH	During a bout (nitroglycerine-induced attacks)	PET	Inferior hypothalamic grey matter activation ipsilateral to the headache side. Increased rCBF in the contralateral ventroposterior thalamus, the anterior cingulate cortex, and in the insulae bilaterally
Sprenger et al. [31]	1 chronic CH	During a bout (spontaneous attacks)	PET	Inferior hypothalamic grey matter activation. Increased rCBF in the medial thalamus and contralateral perigenual ACC
May et al. [32]	17 episodic CH	9 during a bout 8 outside of a bout (nitroglycerine-induced attacks) 1 spontaneous attack	PET MRA	Activation ACC bilaterally, ipsilateral posterior thalamus, ipsilateral basal ganglia, ipsilateral inferior posterior hypothalamus, frontal lobes, insulae bilaterally, contralateral inferior frontal cortex. Increased CBF in the internal carotid artery ipsilateral to the headache side, both in CH patients and in experimentally induced pain
Sprenger et al. [4]	11 episodic CH patients	In- and outside of a bout	FDG-PET	Increased metabolism in the perigenual ACC, posterior cingulate cortex, the orbitofrontal cortex including the nucleus accumbens, ventrolateral prefrontal cortex, DLPFC and temporal cortex, cerebellopontine area. Hypometabolism in the perigenual ACC, prefrontal and orbitofrontal cortex
Lodi et al. [34]	18 episodic CH 8 chronic CH	10 in- and 8 outside of a bout	^1^H-MRS	Reduction of NAA in the hypothalamus of all the patient groups
Wang et al. [35]	47 episodic CH	In- and outside of a bout	^1^H-MRS	Reduction of NAA and Cho/Cr metabolite ratio in the hypothalamus
May et al. [36]	25 episodic CH	In- and outside of a bout	VBM	Increase in bilateral hypothalamic gray matter volume
Sprenger et al. [6]	6 episodic CH 1 chronic CH	During the bout, but out of an acute attack	PET with the opioidergic ligand [11C]diprenorphine	Decreased tracer binding in the pineal gland
Morelli et al. [39]	4 episodic CH	Inside the bout during an acute attack	fMRI	Activation of hypothalamus, pre-frontal cortex, anterior cingulate cortex, contralateral thalamus, ipsilateral basal ganglia and the insula and the cerebellar hemispheres bilaterally
Matharu et al. [43]	7 PH	During acute attack-off indomethacin or pain-free-off indomethacin or pain-free due to indomethacin administration	H_2_^15^O PET	Activation in the contralateral posterior hypothalamus, contralateral ventral midbrain, ipsilateral lentiform nucleus, anterior and posterior cingulate cortices, bilateral insulae, bilateral frontal cortices, contralateral temporal cortex, contralateral postcentral gyrus, precuneus, and contralateral cerebellum. Indomethacin administration turned off the persistent metabolic activation observed during acute attack-off indomethacin
May et al. [45]	1 SUNCT	During 6 consecutive attacks	fMRI	Activation in the ipsilateral inferior posterior hypothalamic gray matter
Cohen et al. [47]	2 SUNCT	During the attacks	fMRI	Activation in the inferior posterior hypothalamic gray matter bilaterally
Sprenger et al. [46]	1 probable SUNCT	During the attack	fMRI	Activation in the ipsilateral hypothalamic gray matter, cingulate cortex, insula, temporal cortex, and frontal cortex
Sprenger et al. [48]	1 SUNCT	During attacks induced touching the upper with the lower lip	fMRI	Activation in the hypothalamic gray matter bilaterally

**Table 2:** Synoptic table of neuroimaging studies showing three major observations relevant for the pathophysiology of cluster headache

Method	Posterior hypothalamus activation	Pain neuromatrix involvement	Opiatergic system involvement
SPECT		Di Piero et al. [28]	
PET	May et al. [30] Sprenger et al. [31] May et al. [32] Matharu et al. [43]	Hsieh [29] May et al. [25] Sprenger et al. [31] May et al. [32] Sprenger et al. [4] Matharu et al. [43]	Sprenger et al. [4] Sprenger et al. [6]
MRI	Lodi et al. [34] Wang et al. [35] Morelli et al. [39] May et al. [45] Cohen et al. [47] Sprenger et al. [46–48] Sprenger et al. [46–48]	Morelli et al. [39] Sprenger et al. [46–48]	
VBM	May et al. [36]		

## Neuroimaging in trigeminal autonomic cephalalgias

The group includes cluster headache (CH), paroxysmal hemicrania (PH), and short-lasting unilateral neuralgiform headache attacks with conjunctival injection and tearing (SUNCT). These syndromes differ in attack duration and frequency and present different responses to therapy [21].

### Cluster headache

CH is the most frequent syndrome. The pain is located mainly around the orbital and temporal regions, though any part of the head can be affected. The headache usually lasts 45–90 min but can range between 15 min and 3 h. Typically, this syndrome is characterized by a striking circannual and circadian pattern. There is a clear male preponderance.

Usually headache attacks cluster in time, lasting for 7 days to a year separated by remission periods lasting for months or years (80–90% of patients). Sometimes attacks recur for more than 1 year without remission periods, then becoming chronic (10–20% of patients) [21, 22].

Early neuroimaging studies in cluster headache evaluated cerebral blood flow, using mainly SPECT. This semi-quantitative method has not provided univocal results, since some studies reported an increase, some a decrease and others no differences in cortical blood flow, possibly because of methodological dissimilarities [23–27]. Di Piero et al. [28], aiming to investigate brain response to pain in 7 cluster headache patients out of the bout compared to 12 healthy controls, recorded Xe-133 SPECT during experimentally induced pain by means of a cold water pressor test. They demonstrated less cerebral blood flow modifications in contralateral primary sensorimotor and thalamic regions compared to healthy subjects only when the CPT was performed on the CH side. This led the authors to suggest the possible involvement of central tonic pain mechanisms in the pathogenesis of cluster headache. Hsieh and coworkers performed [^15^O] butanol PET in 7 patients affected by episodic CH (4 in and 3 out of the bout) during nitroglycerine-induced pain. PET scan showed a significantly increased rCBF in the right caudal and rostrocaudal anterior cingulate cortex (ACC), temporopolar region, supplementary motor area, bilaterally in the primary motor and premotor areas, opercular region, insula/putamen, and lateral inferior frontal cortex. Moreover, a reduction in rCBF bilaterally in the posterior–parietal cortex, occipito-temporal region and prefrontal cortex was observed [29]. May and colleagues, scanning nine chronic CH patients with H_2_^15^O PET during nitroglycerine-induced attacks, were the first to clearly demonstrate inferior hypothalamic gray matter activation ipsilateral to the headache side. Moreover, they observed an increased rCBF in the contralateral ventroposterior thalamus, the anterior cingulate cortex, and in the insulae bilaterally as well [30]. Later, other authors confirmed these data in a spontaneous headache attack of a chronic CH patient during an ongoing H_2_^15^O PET study [31]. The CH attack-induced activation also increases in the medial thalamus and contralateral perigenual ACC [31].

The exact role of the intracranial blood vessels in the mechanism of cluster headache was investigated by May et al. [31], who performed a H_2_^15^O PET study in a group of 17 episodic CH patients (9 in the active period and 8 out of their bout) and a MR angiography in a spontaneous CH and capsaicin-induced pain in 4 healthy volunteers. H_2_^15^O PET study showed significant activation during spontaneous or nitroglycerine-induced headaches bilaterally in the ACC, ipsilateral posterior thalamus, ipsilateral basal ganglia, ipsilateral inferior posterior hypothalamus, both frontal lobes, bilaterally in the insulae, and in the contralateral inferior frontal cortex. In addition, they found activation in large intracranial vessels on the PET scan which corresponds to a significantly increased blood flow in the internal carotid artery ipsilateral to the headache side, both in CH patients and in experimentally induced pain [32]. The latter findings further support the neuronal nature of the dysfunction in CH, confirming that intracranial vascular changes are not specific to headache but represent a generic epiphenomenon of the pain.

Sprenger and colleagues [4] measured cerebral glucose metabolism by means of FDG-PET in 11 episodic CH patients during and outside the bout and compared these patients with a group of 11 healthy subjects. With respect to those outside the bout, patients scanned during the bout presented increased metabolism in the perigenual ACC, posterior cingulate cortex, the orbitofrontal cortex including the nucleus accumbens, ventrolateral prefrontal cortex, dorsolateral prefrontal cortex (DLPFC) and temporal cortex, and increased metabolism in cerebellopontine area. Moreover, CH patients (in and out of the bout) compared to healthy subjects revealed hypometabolism in the perigenual ACC, prefrontal and orbitofrontal cortex [4]. Interestingly, the perigenual ACC was found to be activated in chronic CH patients, unresponsive to pharmacological therapy, who were treated successfully with occipital nerve stimulation [33].

Further evidence for hypothalamic dysfunction in CH arises from spectroscopic studies. A study with proton MR spectroscopy (^1^H-MRS) of 26 patients with CH (18 episodic, 10 in and 8 outside the bout, and 8 chronic) demonstrated that the NAA, a marker of neuronal integrity, is reduced in the hypothalamus of three subgroups when compared to 12 healthy subjects [34]. These data have been confirmed with the same methodology in a group of 47 episodic CH patients by Wang and colleagues [35], who found in addition, a reduction in the Cho/Cr metabolite ratio, both during and out of the bout. This suggests that the hypothalamus in cluster headache might be characterized not only by a neuronal dysfunction but even by changes in the membrane lipids.

With the voxel-based morphometric (VBM) analysis, May and coworkers [36] studied 25 episodic patients with CH and reported an increase in bilateral hypothalamic gray matter volume, with similar results in patients examined during and outside the bout [36]. Another larger VBM study investigating 75 CH patients (22 episodic inside bout, 35 outside bout and 18 chronic CH) was unable to reveal changes in hypothalamic area neither overall nor in subgroups [37].

VBM-MRI findings taken together with those provided by ^1^HMRS may indicate that the hypothalamus of patients with CH has an increased neuronal density with reduced NAA, suggesting the presence of either immature or dysfunctional neurons [34, 38]. Moreover, these morphometric and functional changes seem to be a permanent disease-related dysfunction since they are not due to the CH history or the cluster phase [34, 36].

Using PET with the opioidergic ligand [11C]diprenorphine in 7 CH patients (6 episodic and 1 chronic) who are in bout but out of an acute attack, Sprenger and colleagues demonstrated a decreased tracer binding in the pineal gland, but not in any other brain structure commonly claimed to be involved in cluster headache pathophysiology [7, 36]. Furthermore, the authors found an inverse relationship between the duration of cluster headaches and opioid receptor binding in the ipsilateral hypothalamus and bilateral cingulate cortices. The latter observation suggests that descending opioidergic mechanisms in the pineal gland and hypothalamus might be involved in the generation of cluster headache attacks.

The study of Morelli et al. [39] is the only one performing fMRI in order to investigate the pattern of cerebral activation during an attack of CH in four episodic patients, showing activation of diencephalic regions, mainly the hypothalamus. Additionally, they documented trends of activation in cerebral areas involved in pain processing (prefrontal cortex, anterior cingulate cortex, contralateral thalamus, ipsilateral basal ganglia and the insula and the cerebellar hemispheres bilaterally) [39].

### Paroxysmal hemicranias

PH is a relatively rare syndrome and the clinical features are highly characteristic [40–42]. Patients typically have unilateral, brief, severe attacks of pain, localized over the first division of the trigeminal nerve, associated with cranial autonomic features that recur several times per day. The duration of the pain is between 2 and 30 min with a frequency of between 1 and 40 attacks per day. It is more common in women and in chronic form. PH responds in a dramatic and absolute fashion to indomethacin, hence the importance of distinguishing it from CH and SUNCT, which are not responsive to indomethacin. Activation of the hypothalamic gray matter during attacks was also observed in paroxysmal hemicranias. Matharu and coworkers [43] were the first to perform H_2_^15^O PET in seven patients affected by PH, scanned during acute attack-off indomethacin or pain-free-off indomethacin and pain-free due to indomethacin administration. The regions significantly activated during headache-off indomethacin versus the pain-free phase were the contralateral posterior hypothalamus, contralateral ventral midbrain, ipsilateral lentiform nucleus, anterior and posterior cingulate cortices, bilateral insulae, bilateral frontal cortices, contralateral temporal cortex, contralateral postcentral gyrus, precuneus, and contralateral cerebellum [43]. Interestingly, indomethacin administration turned off the persistent activation observed during acute attack-off indomethacin. This study supports the view of paroxysmal hemicrania as a central nervous system disorder and demonstrates for the first time the metabolic correlation with indomethacin efficacy.

### Short-lasting unilateral neuralgiform headache attacks with conjunctival injection and tearing

Among the other TACs, SUNCT is a very rare primary headache syndrome, with a male prevalence, characterized by strictly unilateral, severe, neuralgic attacks centered on the ophthalmic trigeminal distribution in association with both conjunctival injection and lacrimation [21, 44]. The duration of each attack is between 5 and 240 s with a frequency ranging between 3 and 200 per day. Although there are marked differences in the clinical pictures of the trigeminal autonomic syndromes, such as the frequency and duration of attacks and the different approaches to treatment, many of the basic features of SUNCT, such as episodicity, autonomic symptoms, and unilaterality, are shared by other headache types, such as cluster headache and chronic paroxysmal hemicrania suggesting a pathophysiological similarity to these syndromes. By studying 6 consecutive spontaneous attacks of SUNCT with fMRI in a 71-year-old woman, May et al. [45] observed activation during the same scanning session in the ipsilateral inferior posterior hypothalamic gray matter during the attacks, compared with the pain-free state. The same ipsilateral area activation was observed also in a 68-year-old man with an atypical case of TAC resembling SUNCT [46]. Additionally, a clear trend of cingulate cortex, insula, temporal and frontal cortices activation was observed [46]. It has to be noted that activation of the posterior hypothalamus was even bilaterally in the two patients affected by SUNCT in the study by Cohen and colleagues [47] and increases parametrically with increasing levels of pain.

Bilateral posterior hypothalamus activation was also observed in the fMRI study of Sprenger et al. [48] where a 49-year-old male patient was scanned during attacks of SUNCT and pain-free state. Also in this case, multiple activations were observed in brain regions involved in the processing of pain [48].

## Conclusions

The role of imaging in TACs has traditionally been directed at ruling out treatable and reversible etiologies (secondary forms of TAC). However, here we have reviewed several studies which used neuroimaging to better understand the pathophysiology of TACs.

**Fig. 1:**
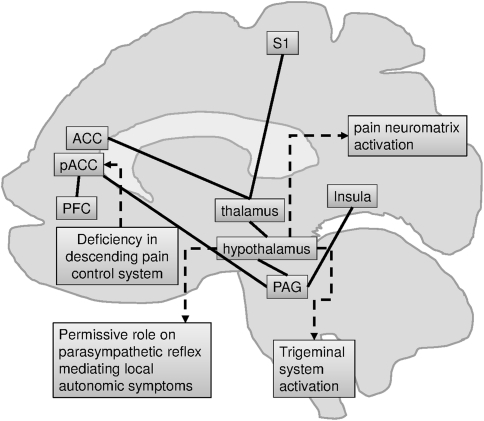
Schematic brain representation summarizing the findings from the previous studies in cluster headache with the possible pathophysiological consequences. *ACC* anterior cingulate cortex, *pACC* perigenual anterior cingulate cortex, *PAG* periaqueductal grey area, *PFC* prefrontal cortex, *S1* primary somatosensory area

Different brain imaging techniques were employed in order to examine the structure, biochemistry, metabolic state, and functional capacity of the TAC brains. All the abnormalities shown by the imaging techniques can be summarized in three major observations: (1) posterior hypothalamic activation during the attacks; (2) involvement of the pain matrix; and (3) of the central opioid system (Fig. 1).

Hypothalamic hyperactivity ipsilateral to the headache side in CH, contralateral in PH, and bilateral in SUNCT were observed during the attacks in all the PET and fMRI studies, with one exception [4]. This activation has not been reported in between attacks of episodic CH. However, hypothalamic activation is not a specific marker of trigeminal autonomic headaches, since it was observed even during spontaneous migraine attacks without aura [49]. It is noteworthy that the presence of hypothalamic activation in other primary headaches or painful syndromes [50, 51] does not necessarily refute specificity for this brain structure in initiating and maintaining an attack of trigeminal autonomic cephalalgia, particularly cluster headache. In fact, hypothalamic activation not only may reflect a general antinociceptive response in healthy humans and in some pathological conditions but may be specifically malfunctioning in cluster headache. As a matter of fact, in patients treated with invasive neurostimulation procedures (occipital nerve stimulation) due to the drug resistance of their chronic cluster headache, the hypothalamic hyperactivation still persists during the stimulator-on condition and despite its clinical efficacy [33]. It has been suggested that persistent hypothalamic activation could be the factor that contributes to generate a central permissive state which predisposes to activation of the trigeminal system, mediating pain, and of the parasympathetic reflex, producing the autonomic symptoms [52]. The latter could also help in explaining why patients still continue to experience autonomic attacks during the neurostimulation-on condition and why pain recurs when it is turned off [33, 54].

For the sake of completeness, it has to be acknowledged that there is still a controversy regarding the exact location of this activation since several authors have questioned whether it reflects a real hypothalamic activation or whether this corresponds to the activation of the ventral tegmental area or of other structures anatomically close to the hypothalamus [55, 56]. The second major finding in trigeminal autonomic headache imaging studies was the involvement of various areas belonging to the so-called pain matrix, such as the prefrontal cortex, anterior cingulate cortex, thalamus, periaqueductal gray, basal ganglia, the insula and the cerebellum. It is noteworthy that these areas are also activated/deactivated in a broad range of painful diseases from visceral to somatic, acute or chronic [51]. For instance, changes in blood flow in the thalamus, insula and anterior cingulate cortex were observed during episodic [57, 58] and chronic [5, 59] migraine attacks, and also in chronic neuropathic [60] and visceral [61] pain. The term pain neuromatrix is used to describe a set of brain regions involved in human nociceptive processing. Moreover, the same brain areas are consistently involved in descending antinociceptive processing. We can thus argue that the metabolic changes in the pain neuromatrix could be considered as the consequence of acute and, more so, chronic pain states like that associated with episodic and chronic cluster headache. This view is reinforced by the fact that the hypermetabolic pattern of most of these brain areas changes, almost restoring normal metabolism, after CCH patients are successfully treated with neuromodulatory procedures [33, 53] or PH patients undergo efficacious treatment with indomethacin [43].

Several implications derive from the observation of hypometabolism in the perigenual ACC (PACC) of episodic CH patients scanned outside, and much more during, the bout compared to healthy subjects [4]. Since PACC seems to play a major role in the central descending opiatergic pain control system, its deficiency may be a mechanism that predisposes to the disorder and to its recurrence [4]. The involvement of the opiatergic system in CH pathophysiology is further confirmed by the same research group who found that opioid receptor availability in the rostral ACC and hypothalamus reduces with the duration of CH [7] and that low-dosage opioid (levomethadone) induces complete and long-lasting CH remission [62]. Moreover, in pharmacologically intractable CCH patients who responded to occipital nerve stimulation, an increased metabolism was observed in PACC in comparison to non-responders [33], further underlining the fact that one of the pathophysiological mechanisms of treatment efficacy in CH is the restoration of normal opioid analgesia.

In conclusion, to date, the most striking neuroimaging findings in cluster headache are the posterior hypothalamic activation during the attacks, with concomitant pain neuromatrix activation and opioid system involvement as underlined by changes in perigenual ACC.

Given the rapid advances in functional and structural neuroimaging methodologies, it can be expected that these non-invasive techniques will continue to improve our understanding into the nature of the brain dysfunction in cluster headache and other trigeminal autonomic cephalalgias.
